# A Manual of Syphilis and the Venereal Diseases

**Published:** 1896-12

**Authors:** 


A MANUAL OF SYPHILIS AND THE VENEREAL DISEASES. By JAMES NEVINS HYDE, A. M., M. D., Professor of Skin and Venereal Diseases, Rush Medical College; Dermatologist to the Presbyterian, Michael Reese and Augustana Hospitals, and Consulting Physician to the Hospital for Women and Children, Chicago; and Frank H. Montgomery, M. D., Lecturer on Dermatology and Genito-Urinary Diseases and Chief Assistant to the Clinic for Skin and Venereal Diseases, Rush Medical College; Attending Physician for Skin and Venereal Diseases, St. Elizabeth Hospital, Chicago. Small 8vo, pp. 618. With forty-four illustrations in the text and eight full-page plates in colors and tints. Price, $2.50. Philadelphia: W. B. Saunders, 925 Walnut street. 1896.

In the introduction of this classic American work, the authors state that, in consequence of the odium associated with venereal diseases, although they were often innocently acquired, this department of medicine became the prey of the charlatan.

There has been a noteworthy change, so that today some of the most cultivated, learned and distinguished physicians are content to labor and glean in the field that was once neglected, abhorred and loathed. Gonorrhea is most often a strictly venereal disorder; syphilis is more frequently a disorder of the innocent; while, as to fatality, gonorrhea in its ultimate results probably destroys more lives than does syphilis.

Both medical historian and archeologist are tracing these maladies back to the remotest antiquity, some even claiming that they originated at the beginning, "When the morning stars sang together." They are certainly hoary with age, although they were not diagnosticated and differentiated as they have been in modern times. Buret says that the venereal virus marked the first steps of human progress along the highway of civilisation, and that syphilis, the daughter of prostitution, was born as soon as commerce, choosing love, presided over the exchange of caresses.

The physician has a sacred duty to perform in the management of these maladies, which bear shame, loathing and remorse, in guarding the secrets intrusted to him, protecting the innocent, conserving the family relation and bringing the sufferer to a successful termination of his disease.

*Acquired Syphilis.*—Syphilis is said to be acquired when transmitted in another way than by inheritance.

*Etiology.*—It has not yet been incontestably demonstrated that microörganisms are effective in the production of this disease. A syphilitic subject surely bears a germ-carrying secretion or virus, which is capable of transmission. When the syphilitic subject ceases to furnish an infectious virus cannot be definitely fixed, although the power to furnish such a virus is gradually lost.

*Chancre.*—Sole and constant characteristics are: (1) An incubative period; (2) a sclerosis, ranging widely in grade and duration in different cases; (3) a simultaneous enlargement and induration of the neighboring gland or glands.

Chancre is a symptom of a syphilis actually present. Chancres in their course mimic any or every cutaneous lesion, including the molecule, papule, vesicle, pustule, bleb, tubercle, tumor and ulcer. The chancre or syphilitic lesion appears at the site of inoculation in from ten to thirty days after infection or inoculation in a vast majority of cases. The mixed chancre at the outset presents all the characteristics of the chancroid, and afterward becomes indurated and develops a syphilitic bubo, followed by the general manifestations of syphilis. Chancres are usually single, but if two or more exist they are multiple from the beginning, unlike chancroids, since the secretion is nonautoinoculable. Induration is constant, varying in degree. Chancres portend the existence of syphilis in some degree. Its severity cannot be prognosticated from the primary local condition. The base of chancroid may be engorged, but not indurated. The bubo of chancroid is usually single and forms abscess. Destruction of the chancre by cautery or excision does not forestall or abort systemic infection, so that mild methods of treatment at this stage of the disease are advised.

As soon as the diagnosis of chancre is made, mercury by the mouth, the one efficient remedy, should be prescribed, with the intent of modifying the local lesion. The various manifestations of this protean malady are fully and elaborately set forth.

*Treatment.*—Hygiene, care and attention to the general health is the first consideration in the management of syphilis. As soon as there is absolute certainty respecting the existence of chancre, begin general treatment, with the understanding that it will not abate or mitigate the symptoms that will follow. A good, early breaking out, vivid and efflorescent, is a favorable augury. Continue treatment for two and a half to three years, at intervals. The manifold manifestations of syphilis are clearly described and many illustrations are given. A most complete and excellent table covering six or seven pages is given, setting forth the diagnostic distinction between chancroid, chancre and the common affections of the genital region.

*Treatment of Syphilis.*—Hygienic care controls in a great measure the gravity of this disease. Trust not exclusively the efficacy of drugs. This malady cannot be aborted or jugulated. General treatment should be instituted as soon as an absolute diagnosis is made. Mercury is given as the sheet-anchor in the early stages and the preparations of iodine in the later periods. The iodine salts are falling into disrepute in the treatment of nervous syphilis and the great value of mercury in the grave complications of this disease is being recognised. The iodine salts are seldom used in the treatment of syphilis at the famous Aachen Springs, Germany. The interesting suggestion is made that syphilitic infection begets, to some degree, immunity to tuberculosis and carcinoma, owing to the inevitable war waged between microörganisms. It is also suggested that the male as well as the female prostitute should be subjected to sanitary rules or laws proposed for preventing the spread of venereal diseases.

The antitoxin serum treatment of syphilis is ignored, although Borling, Hericourt, Richet and others report gratifying success following its use. Syphilitic antitoxic serum is regularly prepared and placed upon the market.

The short chapter on sexual hypochondriasis should be read by every practising physician, as well as every male of hypochondriacal tendencies. Acute urethritis is fully reviewed, concisely and conservatively considered, though nothing new is added. Treatment with the view of aborting gonorrhea and irrigation in the early stages of urethritis are not advised.

*Chronic Urethritis.*—The rôle of the gonococci in the etiology of chronic urethritis is not yet fully determined, and it seems that the pathological processes instituted by the gonococcus continue after the disappearance of the latter. A man with chronic urethritis has no means of knowing whether he may infect or not. Finger says that the conditions of safety are, the absence of gonococci, pus corpuscles and periurethral complications. Noeggerath says that men never fully recover from gonorrhea ; that nine-tenths of the women whose husbands have had gonorrhea eventually develop pelvic inflammations due to infection. The twin authorship, referring to the treatment of posterior urethritis, advise reduction of the inflammation by deep irrigation, using a short tube and hydrostatic pressure, obtained by raising the reservoir to a sufficient height to overcome the compressor muscle by the force of gravity. In the experience of the reviewer this is often a painful and ineffectual process. Bauer says that so long as urine is passed with pathological force, posterior urethritis cannot be cured. If this be so, are not forced injections also harmful ? There is a forceless way of irrigating the bladder and deep urethra.

The principle that hot saline solutions produce rhythmic action (see experiments of Isaac Ott with the bladder of a cat,) may be used for irrigating the urinary tract and thus avoid the traumatism of forced injections as well as that by a catheter. The presence of saline solution at a temperature of 90° or more, with the body postured as described in articles printed in the BUFFALO MEDICAL JOURNAL, March, 1893, and September, 1895, will cause a rhythmic or vermicular action of the urethra, so that the fluid is passed into the bladder without any more force than sufficient to dilate the anterior urethra. Civiale noted a swallowing action of the urethral canal and Rowden McNamara maintained that under certain conditions there was a vermicular action of the urethra bladderward. Careful attention to the details of this procedure will enable anyone to pass water into the bladder. A smaller return flow is essential, so that the fluid in the tubing shall not chill. Not only this, but a moving current is more insinuating than still water. By this method any man may irrigate the lower urinary tract with relief and comfort at any stage of inflammatory action. Such irrigation prepares the subject of chronic urethritis for the necessary local treatment by instillation and insufflation.

This manual of syphilitic and venereal diseases should be read and studied by every physician. It is an honor to its authors and the American profession. B. H. D.

